# Short-term effects of elexacaftor/tezacaftor/ivacaftor in pediatric cystic fibrosis patients in Brazil: a case series

**DOI:** 10.36416/1806-3756/e20230403

**Published:** 2024-11-16

**Authors:** Marta Amor-Barbosa, Fernanda Maria Vendrusculo, Matias Epifanio, Marcio Vinicius Fagundes Donadio, Leonardo Araujo Pinto

**Affiliations:** 1. Departament de Fisioteràpia, Universitat Internacional de Catalunya, Barcelona,Espanya.; 2. Pontifícia Universidade Católica do Rio Grande do Sul, Porto Alegre, Brazil.

## TO THE EDITOR:

Cystic fibrosis (CF) is a chronic autosomal recessive disorder, with an estimated incidence of approximately 1 in 7,000 live births.^1^ It results from genetic mutations on chromosome 7, affecting the CF transmembrane conductance regulator (CFTR) protein.^2^ A deeper understanding of the molecular consequences of mutations in the *CFTR* gene has led to the development of small-molecule modulators, such as the triple combination of elexacaftor, tezacaftor, and ivacaftor (ETI), which enhance CFTR activity and improve organ function in patients with CF.^3^ CFTR modulators are transforming the lives of CF patients, with short- and long-term clinical improvements.^4^ ^,^ ^5^ Real-life studies in Brazil have detailed the remarkable effects that ETI therapy has on adult CF patients.^6^ Nevertheless, to our knowledge, this is the first case series showcasing the short-term effects of ETI therapy on pediatric patients in Brazil, all of whom were monitored at a CF referral center in southern Brazil. Written informed consent was obtained from the patients for publication of the details of their medical case and any accompanying images. 

**Figure 1:**
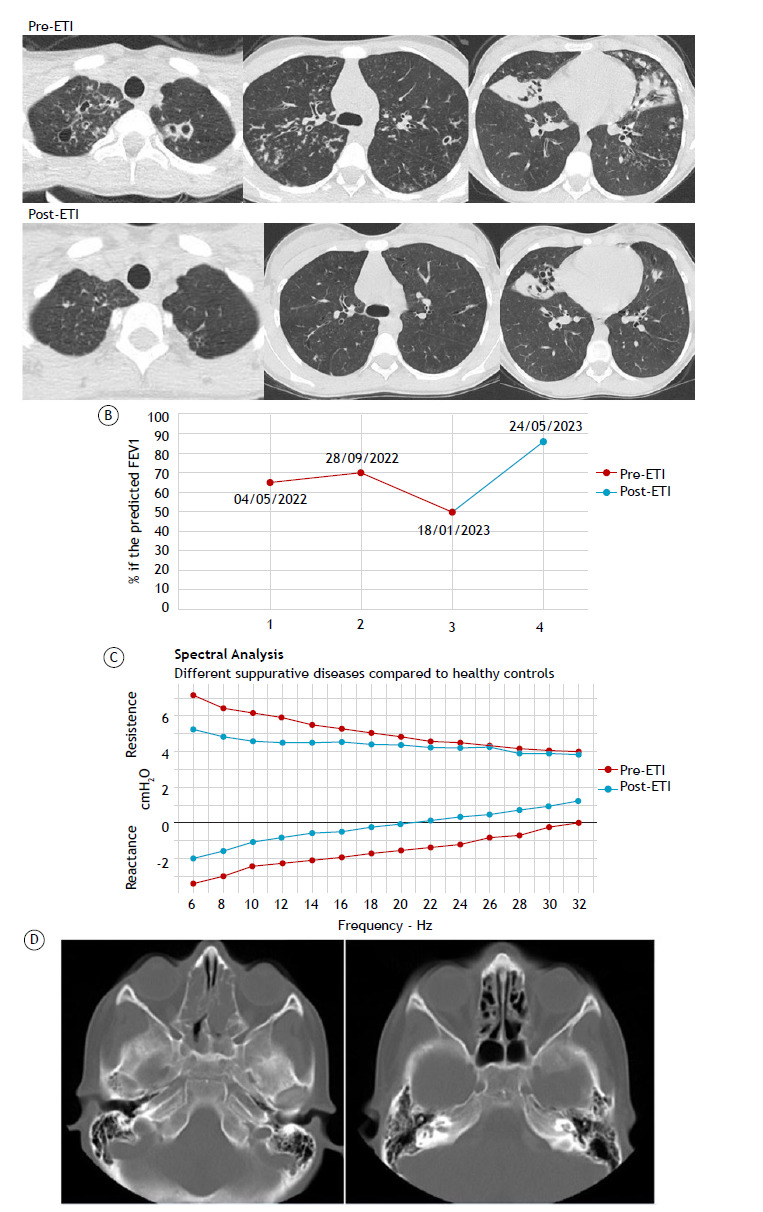
Short-term effects of the triple combination of elexacaftor, tezacaftor, and ivacaftor (ETI) on pediatric cystic fibrosis patients in Brazil. In A, B, and C, bronchiectasis, lung function, and oscillometry in a 12-year-old Caucasian female diagnosed with a homozygous *F508del* cystic fibrosis transmembrane conductance regulator (*CFTR*) gene mutation. In A, note significant improvement in bronchiectasis, as indicated by a decrease in the Brody score (from 78 before initiation of ETI therapy [left] to 18 at 12 months after initiation of ETI therapy [right]). In B, note an increase in FEV_1_ after initiation of ETI therapy. In C, note a reduction in resistance and reactance between the measurements taken before and after initiation of ETI therapy. In D, a CT scan of the nose and sinuses of a 10-year-old Caucasian male diagnosed with heterozygous *F508del*/*R1162X CFTR* gene mutation. Note bilateral soft tissue density polyps before initiation of ETI therapy (left). At 8 weeks after initiation of ETI therapy, the nasal polyps had completely regressed (right).

The first case report shows remarkable short-term lung function improvements in a 12-year-old Caucasian female with homozygous *F508del* mutation in the *CFTR* gene. She had pancreatic insufficiency, no liver impairment, bronchiectasis (a Brody score of 78; Figure 1A), and chronic *Pseudomonas aeruginosa* colonization. On January 18, 2023, her BMI was 15.2 kg/m^2^ (her z-score was −1.31), and spirometry revealed impaired lung function: an FVC of 1.52 L (60%), an FEV_1_ of 1.11 L (50%), an FEV_1_/FVC ratio of 73%, and an FEF_25-75%_ of 0.88 L/min (31%). On April 5, 2023, she was started on treatment with ETI, being reevaluated on May 24, 2023. Although she reported significant improvement in respiratory and gastrointestinal symptoms, she still had pancreatic insufficiency, with no change in fecal elastase levels. She also showed a slight increase in her BMI, to 16.4 kg/m^2^ (her z-score was −0.79), which does not indicate a notable improvement in nutritional status. Spirometry results showed normal lung function: an FVC of 2.55 L (95%), an FEV_1_ of 2.05 L (86%), an FEV_1_/FVC ratio of 88%, and an FEF_25-75%_ of 1.96 L/min (64%). Treatment with ETI effectively reversed lung function decline, with FEV_1_ increasing by more than 35%. Figure 1B depicts FEV_1_ improvement from the year before initiation of ETI therapy. Oscillometry showed improvements in lower airway obstruction (Figure 1C). At 12 months after initiation of ETI therapy, a CT scan showed a remarkable improvement in bronchiectasis (a Brody score of 18; Figure 1A). 

The second case report shows complete regression of nasal polyps in a 10-year-old Caucasian male with a heterozygous *CFTR* gene mutation (*F508del*/*R1162X*). He had pancreatic insufficiency, no liver impairment, normal lung function, and no bronchiectasis (a Brody score of 24). He had a history of nasal obstruction and polyps, which required surgical removal-performed on August 4, 2021 and subsequently on August 1, 2022. During a follow-up evaluation performed on January 18, 2023, a CT scan showed that nasal polyps had reappeared (Figure 1D). His BMI was 15.3 kg/m^2^ (his z-score was −0.92). Spirometry results showed normal lung function: an FVC of 2.60 L (101%), an FEV_1_ of 2.27 L (104%), an FEV_1_/FVC ratio of 94%, and an FEF_25-75%_ of 2.78 L/min (112%). The patient was started on treatment with ETI on April 22, 2023 and underwent a follow-up evaluation on May 25, 2023. Spirometry results continued to show normal lung function: an FVC of 2.73 L (105%), an FEV_1_ of 2.47 L (112%), an FEV_1_/FVC ratio of 91%, and an FEF_25-75%_ of 3.25 L/min (130%). He reported a substantial improvement in nasal obstruction and congestion, the absence of nasal polyposis being confirmed by a CT scan performed on June 23, 2023. Remarkably, within a few weeks of ETI initiation, the nasal polyps had completely regressed (Figure 1D). 

The third case report describes significant clinical improvements in a 14-year-old Caucasian female with heterozygous *N1303K*/*1078del* mutations in the *CFTR* gene. Notably, these mutations have yet to be approved for triple combination therapy with ETI in some countries. She had pancreatic insufficiency, no liver impairment, bronchiectasis (a Brody score of 36), and chronic *Burkholderia cepacia* complex colonization. On March 8, 2023 she presented with a BMI of 20 kg/m^2^ (her z-score was 0.15), and spirometry showed a slight decrease in lung function: an FVC of 2.25 L (80%), an FEV_1_ of 1.99 L (79%), an FEV_1_/FVC ratio of 88%, and an FEF_25-75%_ of 3.15 L/min (98%). She was started on treatment with ETI on July 6, 2023 (provided by the family), and a follow-up evaluation performed on August 9, 2023 revealed significant respiratory symptom improvement (as reported by the patient). Spirometry showed normal lung function: an FVC of 2.74 L (95%), an FEV_1_ of 2.44 L (94%), an FEV_1_/FVC ratio of 89%, and an FEF_25-75%_ of 3.67 L/min (111%). Treatment with ETI effectively reversed lung function decline, resulting in substantial spirometry increases: in FVC by 0.49 L (15%), in FEV_1_ by 0.45 L (15%), in FEV_1_/FVC by 1%, and in FEF_25-75%_ by 0.52 L/min (13%). 

In conclusion, this case series represents the first study of the short-term effects of ETI therapy on pediatric CF patients in Brazil. The cases reported here show the positive impact of ETI on different clinical parameters, including reduced patient-reported symptoms, improved lung function, and complete nasal polyp regression. Moreover, none of the patients experienced any adverse effects of ETI, a finding that corroborates the positive safety profile of ETI.^5^ The findings of the present study are consistent with those of a study showing improvements in the lung function of adult CF patients in Brazil^6^ and provide initial documentation of nasal polyp regression in a pediatric patient. Furthermore, the clinical benefits observed in a patient with the *N1303K* mutation are consistent with previous in vitro and clinical data,^7^ ^,^ ^8^ showing significant benefits from triple combination therapy with ETI in CF patients with this mutation. 
